# Salivary microbiome in children with Down syndrome: a case-control study

**DOI:** 10.1186/s12903-022-02480-z

**Published:** 2022-10-06

**Authors:** Seiji Morishima, Kaori Takeda, Setsue Greenan, Yoshinobu Maki

**Affiliations:** The Lion Foundation for Dental Health, 1-3-7, Honjo, Sumida-ku, Tokyo, 130-8644 Japan

**Keywords:** Down syndrome, Saliva, Microbiota, Children

## Abstract

**Background:**

Down syndrome (DS), a most frequently occurring genetic disorder, is associated with oral morphological abnormalities and higher incidence rates of oral diseases. Recent studies have analyzed the oral microbiome to elucidate their relationships with oral diseases and general health; however, reports on the oral microbiome in individuals with DS are scarce. This study aimed to characterize the oral microbiome in children with DS.

**Methods:**

A total of 54 children aged 1–13 years were enrolled in this case-control study. Of these children, 27 had DS (Case: DS group) and 27 were age-matched healthy children (Control: ND group). Saliva in the oral cavity was collected with a swab, cultured, and tested for cariogenic and periodontopathic bacteria by quantitative polymerase chain reaction (qPCR) detection, and the salivary microbiome was analyzed using next-generation sequencing. The student’s *t*-test, Fisher’s exact test, Mann–Whitney *U* test, and permutational multivariate analysis of variance were used for statistical analysis.

**Results:**

Results of culture and qPCR detection tests for cariogenic and periodontopathic bacteria showed no significant differences in the detected bacteria between the DS and ND groups, with the exception of a significantly higher detection rate of *Candida albicans* in children with DS with mixed dentition. A comparison of the salivary microbiomes by 16S sequencing showed no significant difference in α diversity; however, it showed a significant difference in β diversity. Children with DS had a higher relative abundance of *Corynebacterium* and *Cardiobacterium*, and lower relative abundance of TM7.

**Conclusions:**

This study provided basic data on the salivary microbiome of children with DS and showed the microbiological markers peculiar to children with DS. However, further research to identify the relationship with oral diseases is warranted.

**Supplementary Information:**

The online version contains supplementary material available at 10.1186/s12903-022-02480-z.

## Background

Down syndrome (DS), also known as trisomy 21, is the most common congenital genetic disorder as well as the most common cause of cognitive disorder in humans that affects approximately 12.6 in 10,000 births in the United States (2006–2010) [1], and 12.3 in 10,000 births in England (2013) [2]. The reported incidence in Japan is 22.6 per 10,000 births according to a 2016 survey, which is approximately twice as high as the incidence rates in Western nations [3]. Regarding dental traits, DS is characterized by abnormal shapes or number of teeth, such as congenitally missing, peg-shaped, conic teeth, or teeth with short roots [4–6]. Furthermore, it has been reported that subjects with DS develop periodontal diseases early and have a rapid progression, and conversely, they have fewer dental caries [7, 8].

Periodontal diseases and dental caries are both bacterial infections, and many previous microbiological studies using methods, such as culturing, polymerase chain reaction (PCR), and DNA–DNA hybridization, have reported differences between subjects with DS and healthy individuals in periodontal pathogens and *Streptococcus mutans* colonization in the oral cavity [9–11].

Recent studies have reported that dental caries and periodontal diseases are bacterial infections caused by *S. mutans* and the “red complex” as keystone pathogens and associated dysbiosis of the oral microbiome [12–14]. Furthermore, advances in 16S high-throughput sequencing have allowed for exhaustive analysis of the microbiome, which has also led to a wealth of new studies on the relationship between localized diseases and general health.

Studies of Down syndrome have also reported a comparisons of microbiomes found in the gut and oral rinse samples between adults with DS and non-DS controls have been reported [15, 16].

The oral cavity is almost sterile at birth. Different oral bacteria are known to colonize and continue to evolve depending on various factors, such as host and environmental factors, including vertical transmission. In particular, *S. mutans* infection that occurs between 19 and 31 months after birth, also known as the “window of infection,” is associated with the onset of dental caries [17].

Moreover, diversity of the oral microbiome is known to grow rapidly with tooth eruption, and it is reported that the microbiome on the dorsal surface of the tongue or in the subgingival plaque stabilizes in around 2 years [18, 19]. However, some other reports have documented that the oral microbiome continues to evolve after 2 years of age [20, 21], thereby leaving many unanswered questions. Colonization of strictly anaerobic gram-negative bacteria, which particularly have strong associations with periodontal disease, is believed to increase with age up until puberty.

Researching the characteristics of the oral microbiome from infancy to childhood is thus important for understanding the microbiome forming process in DS. This case-control study compared the differences in salivary microbiomes between children with and without Down syndrome by detecting the pathogenic microorganisms using culture and quantitative PCR methods and high-throughput sequencing.

## Methods

### Participants and ethical review

The participants of this case-control study were children with Down syndrome who lived in Tokyo and were recruited from the Lion Foundation for Dental Health Tokyo Dental Clinic. Twenty-seven children who met the inclusion criteria were included in the study as case group patients. The 27 age-matched healthy controls enrolled were recruited among 15 pupils of a nursery school in Tokyo and 138 students of an elementary school also in Tokyo. The enrolled participants were further divided into children in the primary dentition (PD) and mixed dentition (MD) to form four groups (Table 1).

**Table 1:** Characteristics of participants enrolled in this study and oral hygiene habits surveyed via questionnaire

	PD stage			MD stage		
	DS (n = 12)	ND (n = 12)	*p* value	DS (n = 15)	ND (n = 15)	*p* value
Age (years)^a^	2.6 (1.3–4.3)	2.4 (1.3–4.4)	NS	10.0 (8.0–13.5)	9.6 (7.8–11.5)	NS
% of boys^b^	58.3	41.7	NS	46.7	46.7	NS
Number of teeth (total)^a^	14.3 (2–20)	17.3 (14–20)	NS	23.3 (19–27)	23.5 (20–26)	NS
Number of deciduous teeth^a^	14.3 (2–20)	17.3 (14–20)	NS	10.5 (1–18)	9.5 (2–17)	NS
Number of permanent teeth^a^	–	–	–	12.7 (5–26)	14.0 (7–24)	NS
Daily frequency of TB^b^	1.8 (0–3)	1.7 (1–3)	NS	2.1 (1–3)	2.6 (1–3)	NS
% children undergoing TB by a parent^b^	100	100	NS	80	33.3	0.008
% children using toothpaste^b^	41.7	58.3	NS	46.7	80	NS
% children using dental floss^b^	25	25	NS	14.3	13.3	NS
% children who visited a dentist in the past year^b^	50	58.3	NS	66.7	73.3	NS
Intake of sweets and sweetened drinks three or more times daily^b^	16.7	8.3	NS	20	6.7	NS

A total of 54 participants were divided into 24 in the primary-dentition stage (PD) and 30 in the mixed-dentition stage (MD), and the PD or MD stage was compared between DS and ND groups

*DS* down syndrome, *ND* non-down syndrome, *TB* tooth brushing, *NS* not significant

The a student’s *t*-test and b Fisher’s exact probability test were performed, wherein *p* < 0.05 was considered statistically significant

Children with standard trisomy 21 were selected for this study, and children who took antibiotics within 7 days were excluded; however, complications of DS were not listed in the exclusion criteria.

This study was approved by the ethics committee of the Japanese Society for Oral Health and was conducted according to their guidelines (Approval no.: 27-4, 27-9). This study was conducted from January 2016 to May 2017, and written informed consent was obtained from the parents of all participants.

### Salivary collection and questionnaire survey

Saliva in the oral cavity was collected using SalivaBio Infant's Swab (SIS: Salimetrics, Carlsbad, CA). The SIS was then washed in 2 mL of sterilized saline solution (Otsuka Pharmaceutical Co., Ltd., Tokyo, Japan) and collected, and the aliquot was centrifuged at 16,400 g for 3 min to obtain the bacterial pellet. Saliva was collected at least 1 h after tooth brushing, eating, or drinking. Before collection of saliva, the parents of the participants were asked to fill out a questionnaire in order to survey the oral hygiene habits of the participants. The oral cavity was inspected for the number of erupted teeth, but parameters such as the severity of plaque or gingival inflammation were not assessed.

### DNA extraction

DNA was extracted from the bacterial pellet using nexttec™ 1-Step DNA Isolation Kit (nexttec Biotechnologie GmbH, Leverkusen, Germany) according to the manufacturer’s instructions, and stored at − 40 °C until it was used for quantitative PCR or next-generation sequencing analysis.

### Quantification of cariogenic and periodontopathic bacteria

Cariogenic and periodontopathic bacteria (*Streptococcus mutans*, *Streptococcus sobrinus*, *Prevotella intermedia*, *Tannerella forsythia*, *Treponema denticola*, and *Porphyromonas gingivalis*) were measured by quantitative PCR. Premix Ex Taq™ Probe qPCR (Takara Biomedicals, Shiga, Japan) was used, and 1 μL of the template DNA was amplified in a 20-μL system containing the 200 nM probe and primer set [22, 23] (Additional file 1). The cariogenic and periodontopathic bacterial DNA samples used for creating the standard curve were extracted from *S. mutans* ATCC25175, *S. sobrinus* ATCC33478, *P. intermedia* ATCC25611, *T. forsythia* ATCC43037, *T. denticola* ATCC35405, and *P. gingivalis* ATCC33277.

Amplification in PCR with CFX Connect™Real-Time PCR Detection System (Bio-Rad Laboratories, Hercules, CA) was performed under the following conditions: initial denaturation at 95 °C for 30 s, followed by 40 cycles at 95 °C for 15 s, at 58 °C for 30 s, and at 72 °C for 30 s.

Results were analyzed with CFX Manager™ Ver.2.1 software (Bio-Rad Laboratories) and the bacterial count was calculated using the calibration curve created with type strains, wherein ≥ 2 × 10^2^ cells/swab was considered positive.

*Candida albicans* was detected by culture. BD CHROMagar™ Candida medium plates (Becton, Dickinson and Company, Sparks, MD) by a smear of 200 µL of collected and serially diluted SIS, cultured for 3 days in an aerobic condition at 37 °C, and the colony forming units were calculated from the count of grown colonies to find the count of *C. albicans* per the total bacterial count.

### 16S sequencing

16S rRNA sequencing was performed using universal primers (27Fmod and 338R) [24, 25]. Specifically, Ex Taq polymerase (Takara Bio, Shiga, Japan) was used to amplify approximately 20 ng of template DNA with Veriti Thermal Cycler (Life Technologies Japan, Tokyo, Japan) under the following cycling conditions: initial denaturation at 96 °C for 2 min, followed by 25 cycles at 96 °C for 30 s, at 55 °C for 45 s, and at 72 °C for 1 min.

The PCR product was purified with AMPure XP magnetic purification beads (Beckman Coulter, CA, USA) and quantified with Quant-iT PicoGreen dsDNA Assay Kit (Life Technologies Japan). After quantification, mixed samples were prepared by pooling approximately equal amounts of each amplified DNA. Samples were sequenced using a MiSeq Reagent Kit V3 (300 × 2 cycles) and a MiSeq sequencer (Illumina, CA, USA), according to the manufacturer’s instructions.

### Data processing

An analysis pipeline was used for processing the 16S rRNA gene V1–V2 region, as previously reported [26, 27]. Briefly, after multiplexed sequencing of the 16S amplicons, sequences were assigned to samples based on their barcode sequences. Reads with an average quality value < 25, inexact matches to both universal primers, and possible chimeric reads were eliminated. Among high-quality reads, 3,000 reads per sample were randomly chosen and used for the comparative microbiome analysis. We sorted selected reads with the average quality value and grouped them into operational taxonomic units (OTUs) using the UCLUST (v.5.2.32) algorithm with a 97% identity threshold [28]. Taxonomic assignments for each OTU were made by similarity searching against publicly available 16S database using the GLSEARCH program (v.36.3.8 g). The 16S database was constructed from three publicly available databases [29]: Ribosomal Database Project v.10.31, CORE (http://microbiome.osu.edu/ (31 January 2017, date last accessed)), and the reference genome sequence database obtained from the NCBI FTP site (ftp://ftp.ncbi.nih.gov/genbank/ (December 2011, date last accessed)). For assignment at the genus and species levels, sequence similarity thresholds of 96% and 97% were applied, respectively [28]. All high-quality 16S V1–V2 sequences were submitted to the DDBJ/EMBL/GenBank database (Accession number DRA012575).

### Data analysis

The student’s *t*-test and Fisher’s exact test were used for the analysis of the results of the questionnaire survey and detection rates of the pathogenic bacteria. The Mann–Whitney *U* test was used for comparisons of diversity and ratios of component bacteria at the genus and species levels.

The principal coordinate analysis (PCoA) was used to visualize similarities/dissimilarities in microbiome structures from the UniFrac Distance [30]. A permutational multivariate analysis of variance (PERMANOVA) was conducted to compare overall microbiome structures.

Differences at *p* < 0.05 were considered statistically significant. All analyses were performed using R software program (v3.4.3).

## Results

After excluding 2 individuals who met the exclusion criteria, the sample size of the DS group was 27 and that of the age-matched controls (ND) was 27, so these 54 individuals were ultimately enrolled in this study.

As shown in Table 1, the 27 participants comprised 12 participants in the PD group and 15 in the MD group, and DS-ND comparisons were made separately in the PD and MD groups. Children who received any antibiotics within the last 7 days were excluded from this study; however, complications of DS were not included in the exclusion criteria. In the PD and MD groups, 8/12 and 10/15 children, respectively, had complications related to DS; in both groups, the most common complication was heart disease, which affected 6/12 and 7/15 of children with PD and MD, respectively.

There were no differences in the participants’ characteristics with regards to age, sex, or the number of teeth. No significant differences were found in questionnaire survey results on oral hygiene in the PD group. The MD group had similar results, except for the percentage of children having “oral hygiene practiced by the parents,” which was significantly higher in the DS group (Table 1).

We collected the saliva samples of 27 children with DS and 27 age-matched children without DS and compared cariogenic and periodontopathic bacteria as well as the salivary microbiome. Salivary cariogenic and periodontopathic bacteria were detected by quantitative PCR. In the PD group, the detection rates of these pathogenic bacteria were low; *S. mutans, P. intermedia, T. forsythia,* and *T. denticola* were detected in only 1–2 children with DS, while none of the pathogenic bacteria were detected in the ND group. While detection rates of pathogenic bacteria in the MD group were higher than those in the PD group, *S. sobrinus* and *P. gingivalis* were not detected. There were no differences between the children with and without DS in terms of other detected pathogenic bacteria. However, among children with DS, the detection rate for *C. albicans* by culture was significantly higher in the MD group (Table 2).

**Table 2:** Frequency of detecting cariogenic and periodontopathic bacteria in saliva samples

Bacteria	PD stage			MD stage		
	DS (n = 12)	ND (n = 12)	*p* value	DS (n = 15)	ND (n = 15)	*p* value
*S. mutans*	1/12	0/12	NS	4/15	3/15	NS
*S. sobrinus*	0/12	0/12	NS	0/15	0/15	NS
*P. intermedia*	1/12	0/12	NS	3/15	3/15	NS
*T. forsythia*	2/12	0/12	NS	5/15	6/15	NS
*T. denticola*	1/12	0/12	NS	4/15	8/15	NS
*P. gingivalis*	0/12	0/12	NS	0/15	0/15	NS
*C. albicans*	0/12	1/12	NS	11/15	0/15	< 0.0001

Cariogenic and periodontopathic bacteria were detected by qPCR

*C. albicans* was detected by culture

The Fisher’s exact probability test was performed for statistical analysis and *p* < 0.05 was interpreted as a statistically significant difference

*DS* down syndrome, *ND* non-Down syndrome, *PD* primary dentition, *MD* mixed dentition, *NS* not significant

**Fig. 1:**
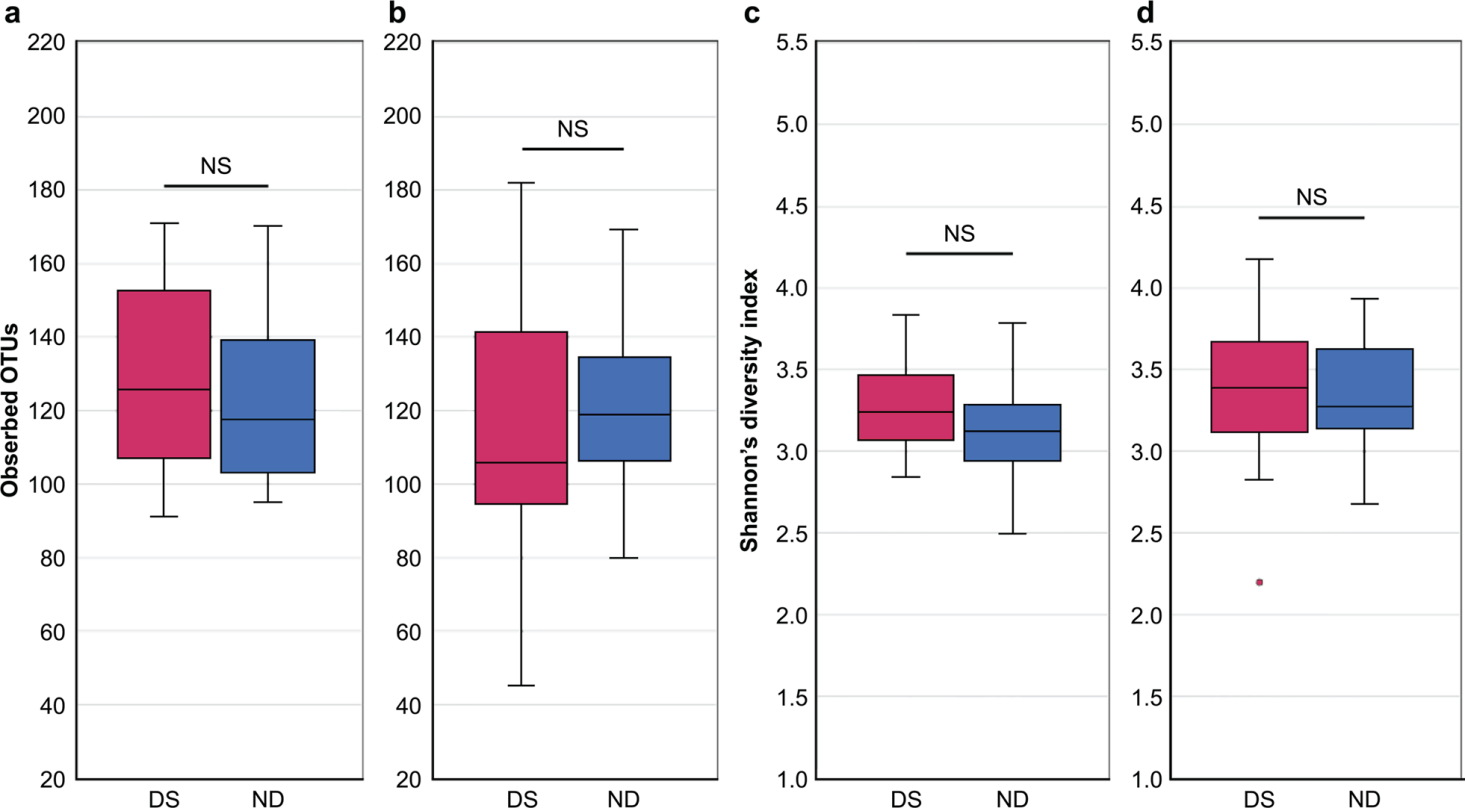
Salivary microbiome α diversity of children with and without DS. Panels **a** and **b** show the OTU counts of detected bacteria. Panels **c** and **d** are box plots of the Shannon index. Panels **a** and **c** compare the primary-dentition stage between children with DS and ND, while panels **b** and **d** compare mixed-dentition stage between children with DS and ND. The Mann–Whitney *U* test was performed for statistical analysis. *p* < 0.05 was considered statistically significant. DS: Down syndrome, ND: Non-Down syndrome, NS: Not Significant

Salivary bacterial microbiomes were compared by high-throughput sequencing. No significant differences were observed between the children with DS and ND in the detected OTU count or Shannon’s diversity index in the PD or MD group (Fig. 1).

**Fig. 2:**
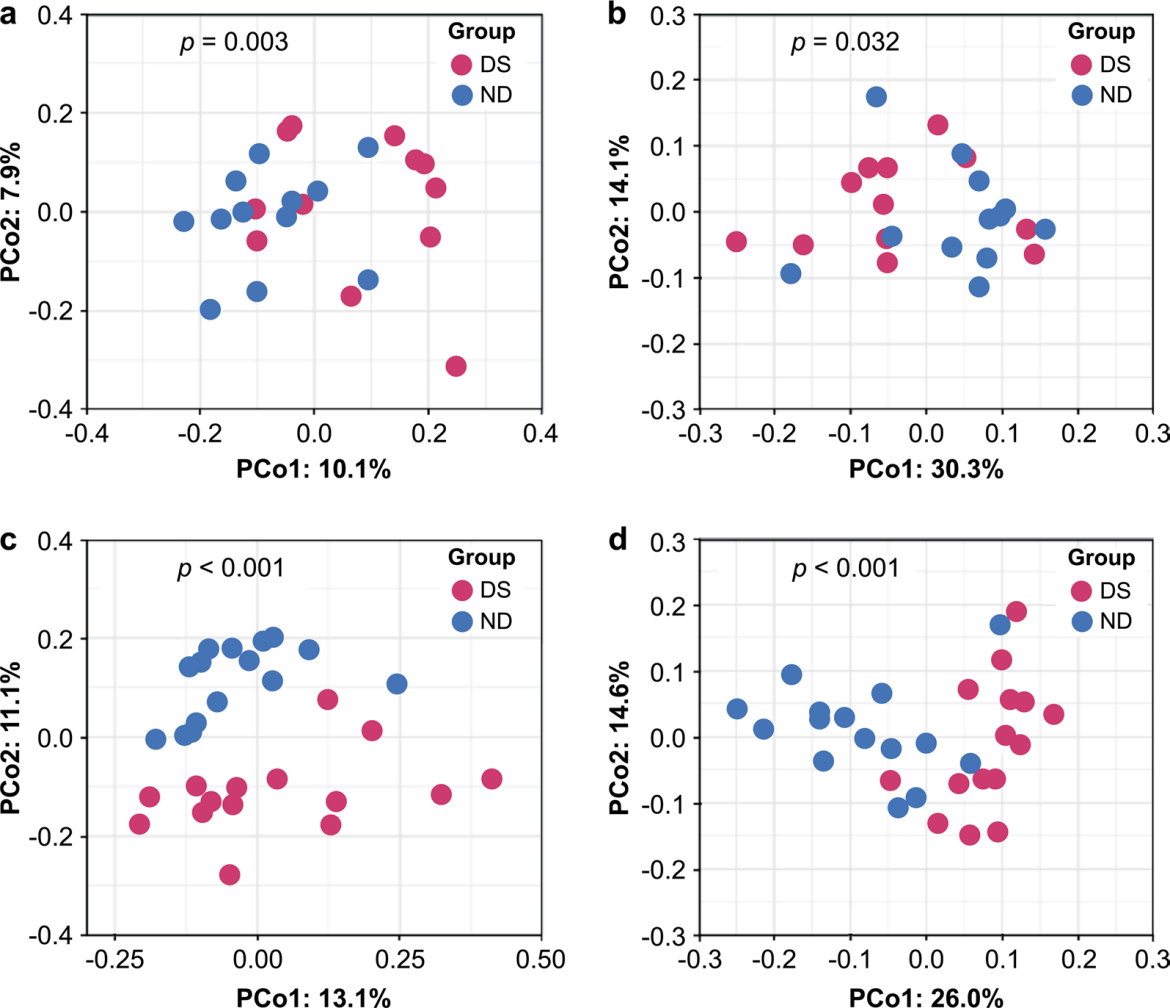
Principal coordinate analysis of microbiome structures in the saliva of children with and without DS. Panels **a** and **c** represent results based on Unweighted UniFrac Distance, while panels **b** and **d** show results based on Weighted UniFrac Distance. Panels **a** and **b** are the data in children with PD stage, and panels **c** and **d** are the data in children with MD stage. Samples of children with DS and ND are shown as red and blue dots, respectively. Statistical analyses were performed by permutational multivariate analysis of variance. DS: Down syndrome, ND: Non-Down syndrome, PD: primary dentition, MD: mixed dentition

Figure 2 displays the results of comparison of similarities/dissimilarities in salivary microbiome between children with DS and ND of the PCoA plot from the UniFrac Distance.

Furthermore, PERMANOVA test showed significant differences in salivary microbiomes in the PD group in weighted and unweighted distances (*p* = 0.003, 0.032). Similarities in the microbiome in the MD group were more noticeable in unweighted and weighted distances (*p* < 0.001).

**Fig. 3:**
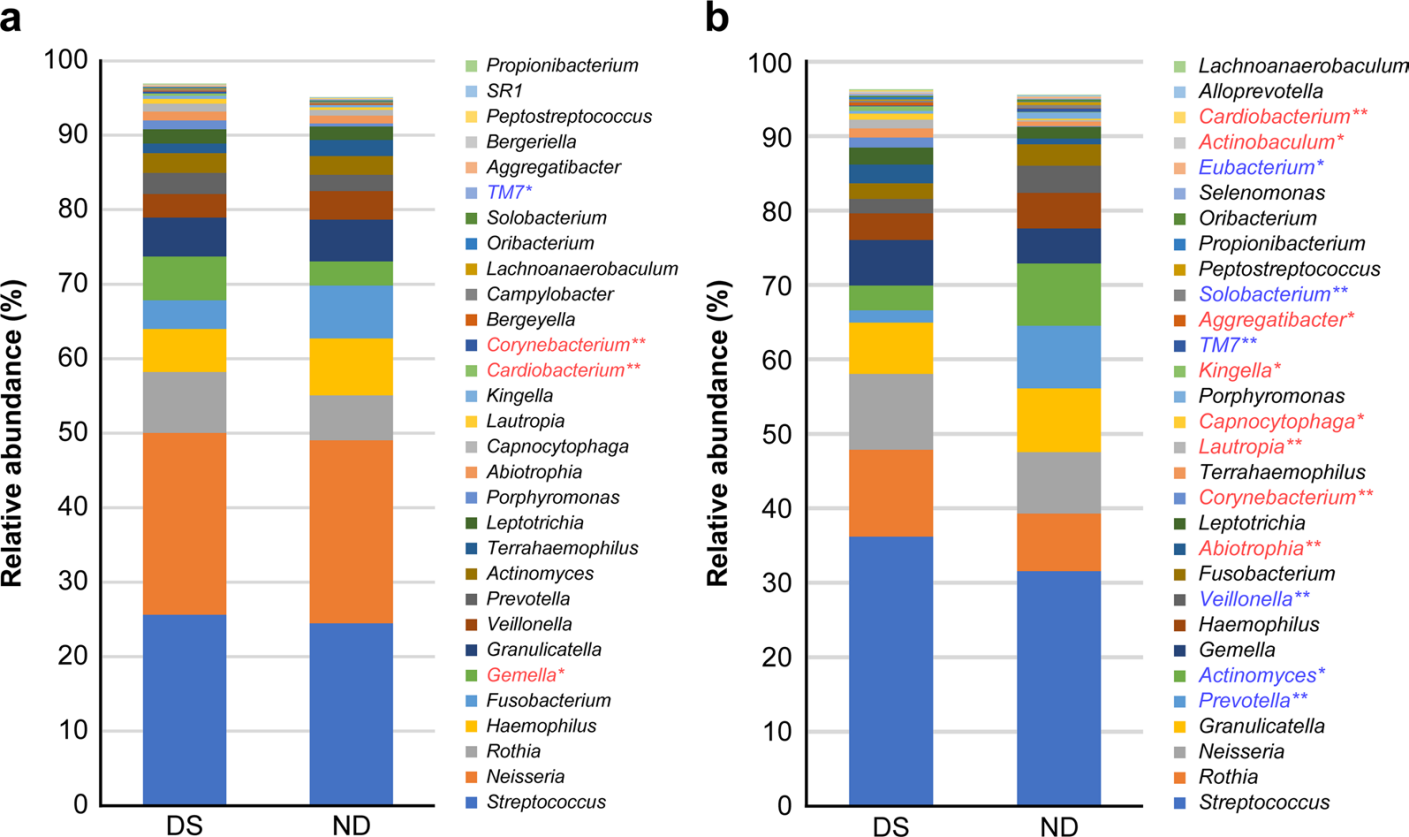
Bacteria composing salivary microbiomes in children with DS and ND (at the genus level). The mean abundance of the top 30 genera composing salivary microbiomes are compared between the group of children with DS and the ND group. The mean abundance (%) in **a** the PD stage and **b** the MD stage are presented. The Mann–Whitney *U* test was performed. Genera in red font were significantly more abundant in DS, and genera in blue font were significantly less abundant in DS (*p < 0.05, **p < 0.01). DS: Down syndrome, ND: Non-Down syndrome, PD: primary dentition, MD: mixed dentition

Figure 3 displays the top 30 genera composing the salivary microbiome of children with DS and ND in the PD and MD stages. Significant differences between children with DS and ND were observed for four genera in the PD group; the relative abundance values of *Gemella, Corynebacterium,* and *Cardiobacterium* were significantly higher in DS, and that of TM7 was significantly lower in DS.

Among the top 30 genera, significant differences were observed for 14 genera in the MD group. In addition to genera *Corynebacterium* and *Cardiobacterium* mentioned above, eight total genera including *Abiotrophia, Lautropia,* and *Capnocytophaga* were also significantly more abundant in DS, whereas a total of six genera including *Prevotella, Actinomyces, Veillonella,* and TM7 mentioned above were significantly less abundant in children with DS in the MD stage.

**Fig. 4:**
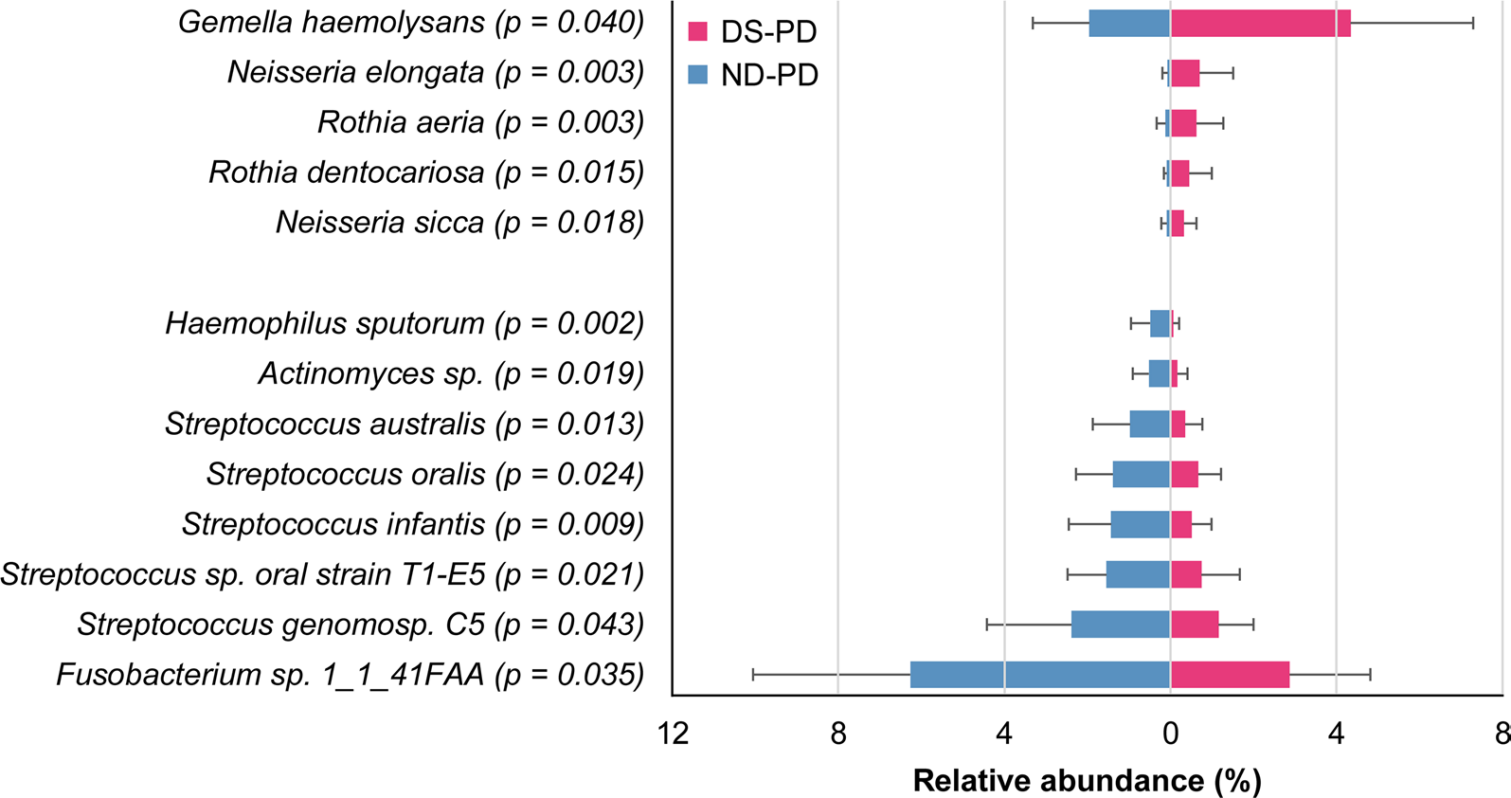
Species level of salivary microbiomes between children with DS and ND (PD stage). The mean abundance (± SD) of 13 species that showed ≥ 0.3% abundance and significant differences. *p* < 0.05% in the Mann–Whitney *U* test was considered statistically significant. DS: Down syndrome, ND: Non-Down syndrome, PD: primary dentition

**Fig. 5:**
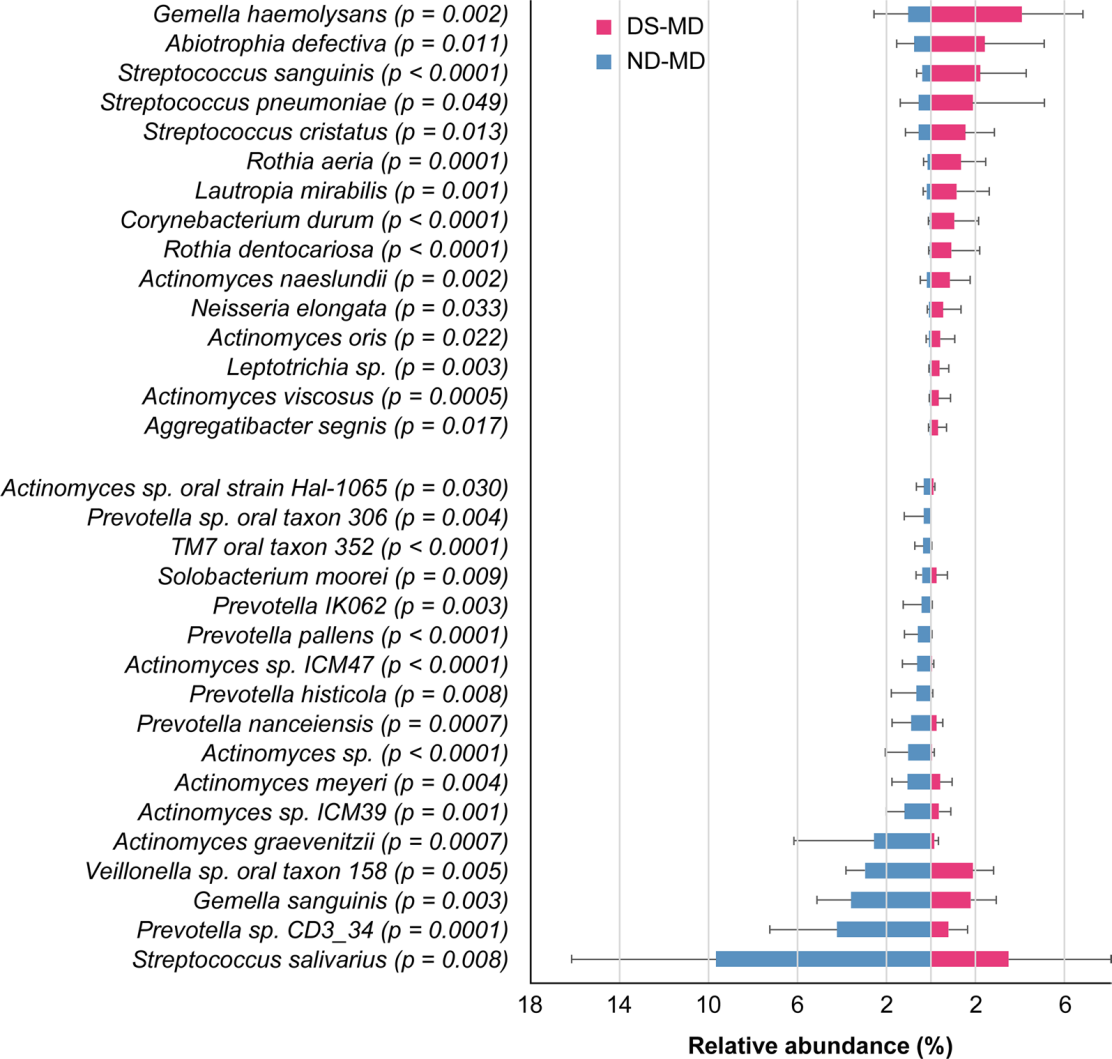
Species level of salivary microbiomes between children with DS and ND (MD stage). The mean abundance (± SD) of 32 species that showed ≥ 0.3% abundance and significant differences. *p* < 0.05% in the Mann–Whitney *U* test was considered statistically significant. DS: Down syndrome, ND: Non-Down syndrome, MD: mixed dentition

Figures 4 and 5 present the bacterial species of which abundance was ≥ 0.3% and differed significantly between children with DS and ND (*p* < 0.05). Significant differences were observed for 13 bacterial species in the PD group and for 32 species in the MD group. *Gemella haemolysans, Neisseria elongata, Rothia aeria*, and *Rothia dentocariosa* were significantly more abundant in children with DS in both the PD and MD groups. Meanwhile, some *Actinomyces sp.* were less abundant in children with DS.

## Discussion

This study compared the salivary microbiomes of children with and without DS aged 1–13 years, which is considered the crucial period for microbiome formation.

Amano et al. and Sakellari et al*.* detected periodontopathic bacteria in 2- to 13-year-old and 8- to 28-year-old subjects with DS, respectively, using quantitative PCR and DNA–DNA hybridization, respectively. They reported that these bacteria were detected earlier in subjects with DS than in healthy subjects [9, 10]. With regards to cariogenic bacteria, Scalioni et al*.* [11] tested for *S. mutans* in the saliva of 3- to 12-year-old children with in situ hybridization, and reported lower detection rates of the species in children with DS than in healthy children.

This study collected the saliva of 27 children with DS and age-matched controls to test salivary cariogenic and periodontopathic bacteria in these samples using quantitative PCR, and found low detection rates of these pathogens in children with PD (aged 1–4 years), and no differences in detection rates between DS and ND in the MD stage (aged 6–12 years) (Table 2).

Studies to date have measured periodontopathic bacteria in the subgingival plaque, whereas this study tested them in saliva; this difference may explain why we did not observe a difference because of a lower detection rate of the red complex, which are composed of strictly anaerobic bacteria. Moreover, rates of colonization of cariogenic bacteria in children in Japan have recently decreased because of the increase in awareness of the importance of oral hygiene [31]; this may have played a role in the difference between the findings of this study and those of the previous studies.

The comparison of bacterial microbiome by 16S rRNA high-throughput sequencing did not reveal any difference between DS and ND in α diversity of the microbiomes in both PD and MD groups; however, a significant difference was observed in β diversity as shown on the PCoA plot (Figs. 1 and 2).

In both PD and MD groups, *Corynebacterium* and *Cardiobacterium* were dominant and TM7 was less abundant among the bacterial genera composing the microbiome in DS. Moreover, there were more genera with significant DS-ND differences in the MD stage than in the PD stage, suggesting that the DS-ND differences in salivary microbiomes may widen with age.

Willis et al. [16] collected the oral rinse samples from individuals with DS aged 7–55 years for a comparison of the microbiome with healthy controls, and reported that the genera *Kingella, Staphylococcus, Gemella, Cardiobacterium, Rothia,* and *Actinobacillus* were more abundant, and *Alloprevotella, Atopobium, Candidatus,* and *Saccharimonas* were less abundant in DS.

The results of this study also showed higher abundance of *Cardiobacterium* in children with DS in both the PD and MD groups, suggesting that the dominance of *Cardiobacterium* is a characteristic of the oral microbiome of DS from a very early stage.

At birth, the oral cavity is practically sterile, while bacterial flora develops under the influence of factors and events, such as mode of delivery, breast or bottle feeding, eruption of tooth, introduction of solids, and oral hygiene status [32, 33]. Among them, the oral hygiene status is one of the major factors of dental caries and onset of periodontal diseases as well as microbiome formation [34].

There are several limitations to this study related to insufficient data collection. First, we could not obtain clinical indices, such as the Oral Hygiene Index and the Gingival Index, and we were unable to study the relationships with the degree of dental plaque accumulation or gingival inflammation. However, the results of the questionnaire survey showed no major differences in the number of teeth or oral hygiene habits (Table 1), suggesting that the differences in the oral microbiome between children with and without DS are primarily due to differences in their oral environments, including salivary properties.

Many characteristics of the saliva of individuals with DS have been reported, including low secretion, increased oxidative stress, increased secretory IgA, and abnormality of inorganic salts [35–39], and all these factors are likely to affect the salivary microbiome [40, 41].

Effects of decreased salivary secretion on the oral microbiome have been discussed in the context of Sjögren’s syndrome and side effects of radiotherapy and drug therapies [42–44]. Such studies have commonly reported higher detection rates of *Lactobacilli* and *C. albicans;* however, the present study showed a low detection rate of *Lactobacilli* and no significant difference between children with DS and ND group. In DS group, the observed high detection rate of *C. albicans* was consistent with the findings of the previous studies.

Overexpression of the superoxide dismutase gene encoded on chromosome 21 has been reported to cause overproduction of hydrogen peroxide and associated hydroxy radicals in subjects with DS than in healthy individuals [45], and accordingly, oxidative stress marker levels in their saliva have been reported to be higher compared to those in healthy individuals [35, 36]. Furthermore, hydrogen peroxide produced by some bacterial species in the genus *Streptococcus* in the oral cavity are known to affect the surrounding microbiome, and hydrogen peroxide produced by *S. sanguinis* inhibits *S. mutans* colonization [46, 47]. Such increased oxidative stress in the saliva may indeed have an effect on the microbiome.

Among the bacterial species found at significantly higher abundance in children with DS in both PD and MD groups, *Neisseria elongate, Rothia aeria*, and *Rothia dentocariosa* are catalase-positive bacteria, except *Gemella haemolysans* [48]. These bacteria are known to show resistance to high oxidative stress environments and may be a factor of dysbiosis in the salivary microbiome of the group of children with DS. Moreover, the aforementioned *C. albicans* also has a catalase gene, which may similarly explain their higher abundance in the DS group.

Dental plaque is formed through cell to cell interactions between bacteria from initial colonizers to the late colonizers in the oral cavity [49], and dental plaque adapted to the environment are known to develop in the supragingival and subgingival plaque [50, 51].

Khocht et al. [52] studied 40 bacterial species in the subgingival plaque of adults with DS using checkerboard DNA–DNA hybridization. They reported that *Streptococcus* sp. (i.e., *S. oralis, S. mitis,* and *S. gordonii*), which are initial colonizers, were more abundant in adults with DS than in healthy individuals. *S. sanguinis*, an initial colonizer, was also significantly more abundant in our participants with DS in the MD stage. These *Streptococcus* bacteria are known to be particularly important in the initial stage of dental plaque formation, and their differences in saliva are thus likely to influence the constituents in subsequent dental plaque formation.

Xiao et al. [53] analyzed supragingival dental plaque in adult patients with dental caries using 16S pyrosequencing and have reported the characteristic presence of *Cardiobacterium* and *Corynebacterium* bacteria in participants without dental caries. Janem et al. [54] studied the salivary microbiome of obese children with and without type 2 diabetes and have shown that *Lautropia, Corynebacterium,* and *Cardiobacterium* bacteria were detected in association with gingivitis.

In this study, the genera *Corynebacterium* and *Cardiobacterium* were dominant in the saliva of children with DS in the PD and MD stages, and the genus *Lautropia* was dominant in children with DS in the MD stage. The observed differences in salivary microbiomes, including the aforementioned differences in *Streptococcus sp.* affect the composition of plaque bacteria and may be associated with the onset of dental caries or periodontal diseases.

These findings suggest that the distinct characteristics of the salivary microbiome in subjects with DS from that in healthy individuals may be attributable to several factors; however, we were not able to measure the amount of salivary secretion or oxidative stress status in this study. Moreover, complications of DS or history of antibiotic administration within 1 week of specimen sampling can also affect the salivary microbiome [55], thus these factors should also be analyzed in future studies to get a better understanding of the dysbiosis of the salivary microbiome in DS. Furthermore, associations between the development of dental caries, periodontal diseases, and bacteria constituting dental plaque in the affected sites in subjects with DS should also be analyzed.

## Conclusions

In conclusion, qPCR and high-throughput sequencing provided basic data on the salivary microbiome in DS and revealed dysbiosis in the salivary microbiome in children with DS when compared with that in children without DS.

## Supplementary Information

**Additional file 1**. Sequences of primers and probes used in the quantitative PCR assays.

## Data Availability

The reported nucleotide sequence data are available in the DDBJ Sequenced Read Archive under the accession numbers DRA012575.

## Abbreviations

DS: Down syndrome
MD: Mixed dentition
ND: Non-Down syndrome
OUT: Operational taxonomic unit
PCoA: Principal coordinate analysis
PCR: Polymerase chain reaction
PD: Primary dentition
PERMANOVA: Permutational multivariate analysis of variance
