# Supplementary material for: Salivary microbiome in children with Down syndrome: a case-control study
Source: BMC Oral Health. 2022 Oct 6;22:438. doi: 10.1186/s12903-022-02480-z (PMC9535924; doi:10.1186/s12903-022-02480-z)
Supplement: Supplementary file 1 — Additional file 1. Sequences of primers and probes used in the quantitative PCR assays. [file 12903_2022_2480_MOESM1_ESM.docx]

Additional file 1: Sequences of primers and probes used in the quantitative PCR assays

|  |  |  |  |
| --- | --- | --- | --- |
| Target Bacteria | Primers and Probe | Sequences 5′**–**3′ | Ref |
| *Streptococcus mutans* | Forward | GCCTACAGCTCAGAGATGCTATTCT | 22 |
|  | Reverse | GCCATACACCACTCATGAATTGA |  |
|  | Probe | FAM-TGGAAATGACGGTCGCCGTTATGAA-TAMRA |  |
| *Streptococcus sobrinus* | Forward | TTCAAAGCCAAGACCAAGCTAGT | 22 |
|  | Reverse | CCAGCCTGAGATTCAGCTTGT |  |
|  | Probe | FAM-CCTGCTCCAGCGACAAAGGCAGC-TAMRA |  |
| *Prevotella intermedia* | Forward | TCCACCGATGAATCTTTGGTC | 23 |
|  | Reverse | ATCCAACCTTCCCTCCACTC |  |
|  | Probe | FAM-CGTCAGATGCCATATGTGGACAACATCG-TAMRA |  |
| *Tannerella forsythia* | Forward | AGCGATGGTAGCAATACCTGTC | 23 |
|  | Reverse | TTCGCCGGGTTATCCCTC |  |
|  | Probe | FAM-TGAGTAACGCGTATGTAACCTGCCCGC-TAMRA |  |
| *Treponema denticola* | Forward | CCGAATGTGCTCATTTACATAAAGGT | 23 |
|  | Reverse | GATACCCATCGTTGCCTTGGT |  |
|  | Probe | FAM-ATGGGCCCGCGTCCCATTAGC-TAMRA |  |
| *Porphyromonas gingivalis* | Forward | ACCTTACCCGGGATTGAAATG | 23 |
|  | Reverse | CAACCATGCAGCACCTACATAGAA |  |
|  | Probe | FAM-ATGACTGATGGTGAAAACCGTCTTCCCTTC-TAMRA |  |
|  |  |  |  |
| PCR: Polymerase chain reaction | |  |  |
